# Maintenance erlotinib improves clinical outcomes of unresectable advanced non-small cell lung cancer: A meta-analysis of randomized controlled trials

**DOI:** 10.3892/etm.2012.690

**Published:** 2012-08-31

**Authors:** JIAN ZHANG, WEIQING ZHANG, SHAOHONG HUANG, HUI LI, YUN LI, HUIGUO CHEN, WEIBING WU, WEI ZHOU, CUIPING WANG, HONGYING LIAO, LIJIA GU

**Affiliations:** Thoracic Surgery Department and Clinical Research Center for Thoracic Oncology, The Third Affiliated Hospital of Sun Yat-sen University, Guangzhou 510630, P.R. China

**Keywords:** non-small cell lung cancer, maintenance therapy, erlotinib, epidermal growth factor receptor, meta-analysis

## Abstract

The aim of this study was to evaluate the efficacy and safety of erlotinib as maintenance therapy in patients with unresectable non-small cell lung cancer (NSCLC) by evidence-based methodology. Six eligible studies including 4,372 patients were analyzed. Erlotinib was administered to 2,191 patients as maintenance treatment, while the remaining patients received a placebo or observation only. The meta-analysis was performed using Reviewer Manager Version 5.12 software. Compared with the control group, maintenance erlotinib improved progression-free survival (PFS) and overall survival (OS) with moderate heterogeneity. Results from the random effects model analysis for OS were not in concordance with the difference observed in the fixed effects model analysis. Administration of erlotinib only after chemotherapy obtained a higher objective response rate (ORR). Safety analyses indicated a slight increase in side-effects. The most common adverse events (AEs) were diarrhea and rash, which were usually manageable. There was no significant difference in treatment-related deaths. Erlotinib produced significant clinical benefits with acceptable toxicity as a maintenance strategy in patients with unresectable NSCLC, particularly when sequentially administered with chemotherapy. However, more well-designed randomized control trials (RCTs) are required to identify patients that may derive greater benefits from maintenance with erlotinib, and whether the use of erlotinib as maintenance therapy is more efficient than second-line treatment should also be investigated.

## Introduction

In the United States, lung cancer is the leading cause of cancer-related mortalities (1). Non-small cell lung cancer (NSCLC) accounts for more than 85% of all lung cancer cases (2). Approximately 40% of lung cancer patients present with advanced NSCLC (1). For patients with advanced NSCLC with a favorable performance status, the first-line treatment is a platinum-based two-drug combination regimen resulting in a slight increase in survival (3). Traditionally, 4–6 cycles of platinum-based chemotherapy are recommended (4) and clinical trials have demonstrated that prolonged first-line treatment did not improve survival but increased toxicity (5–7). As combined chemotherapy is reaching a plateau of efficacy, a ‘watch and wait’ strategy until disease progression was previously considered a reasonable therapeutic strategy. The majority of patients rapidly suffer from disease progression within 2–3 months of their last chemotherapy cycle (8,9).

Second-line treatments are provided to those who experience disease progression after first-line therapies. However, certain studies suggest that 30–50% of patients do not receive effective second-line therapy after disease progression due to rapid progression, declining performance status and increased symptom burden (9–13). Consequently, investigations were conducted to identify a maintenance strategy as a more active therapy, which is expected to consolidate the effectiveness of first-line chemotherapy and improve survival without serious toxicity. More patients with a favorable condition are able to receive this regimen due to lack of disease progression.

Maintenance therapy is defined as the prolongation of treatment duration with the administration of additional drugs at the end of a defined number of initial chemotherapy cycles after achieving tumor control. Maintenance therapy has been extensively investigated in patients with NSCLC. It consists of drugs included in the first-line treatment or other non-cross-resistant agents. Although the exact terminology remains unclear, according to the literature, when drugs included in the induction regimen are used it is known as ‘continuation maintenance’ and when other non-cross-resistant agents are used, it is designated as ‘switch maintenance’ or ‘early second-line’ (13,14). Previously, many agents were extensively explored for maintenance therapy in patients with advanced NSCLC, including cytotoxic drugs, targeted agents and anticancer vaccines. Promising results, including improvements in progression-free (PFS) or overall survival (OS), were reported (8,9,15–17). Erlotinib is a highly potent, orally active epidermal growth factor receptor (EGFR) tyrosine-kinase inhibitor (TKI) approved worldwide for advanced NSCLC treatment following chemotherapy failure. In a large phase III trial, erlotinib monotherapy was proven to significantly improve OS [6.7 vs. 4.7 months; hazard ratio (HR)= 0.70; P<0.001] vs. placebo and provide significant symptom and quality-of-life benefits in patients previously treated with advanced NSCLC (18), which was in agreement with another large phase IV clinical study (19). Expected to benefit more patients, this targeted agent was also studied in several trials to test the efficacy and safety when used concurrently or sequentially with fist-line chemotherapy in patients who obtained disease control. However, the PFS and OS results remain controversial. Two early studies showed that erlotinib did not improve PFS and OS compared with the placebo when it was combined with chemotherapy as first-line treatment for advanced NSCLC (20,21). A multi-centre, randomized, placebo-controlled phase III study suggested that maintenance therapy with erlotinib for NSCLC patients is well-tolerated and significantly prolonged PFS and OS compared with the placebo (16). Similar discrepancies also exist among other studies. Therefore, a meta-analysis was performed to examine pooled data of randomized control trials (RCTs) where erlotinib was compared against placebo or observation only in the maintenance regimen for patients with unresectable NCSLC to quantify potential benefits and determine safety.

## Patients and methods

### Search strategy and study selection

A wide search of the main electronic databases of interest was conducted, including PubMed, EMBASE, the Cochrane Library Trials Register, the National Cancer Institute Clinical Trials, the Clinical Trials Register of Trials Central and the abstracts published in the Proceedings of American Society of Clinical Oncology (ASCO). The reference lists of primary studies and relevant review articles were also examined manually for potential eligible studies. An additional search was performed on the Web of Science database for studies cited if necessary.

The search strategy for PubMed was constructed using a combination of Medical Subject Headings (MeSH) and text words relating to erlotinib or Tarceva^®^ for NSCLC. A total of two searches were performed: i) erlotinib OR Tarceva [All Fields] and ii) (Lung cancer) OR (pulmonary carcinoma) [All Fields] OR (Lung neoplasm) [MeSH Terms]. The final search combined i) and ii) and was limited to humans, clinical trials, meta-analyses, practice guidelines, RCTs and reviews. The search strategies for the other databases were based on keywords similar to the terms described above. The electronic search was conducted until June 2011, without language limitations.

### Inclusion and exclusion criteria

Studies selected from this initial search were subsequently screened for eligibility using the following criteria: i) participating patients with unresectable NSCLC at baseline levels, ii) studies in maintenance therapy with vs. without erlotinib after the first-line chemotherapy and iii) RCTs with parallel design. Studies were excluded based on the following criteria; i) patients previously treated with targeted agents, ii) phase I clinical trial, iii) retrospective trial or iv) any review, comment or case report.

### Selection, assessment and data extraction

To select eligible studies for further evaluation, two independent reviewers screened the title, abstract and keywords of every study retrieved. Full articles were assessed if the study conformed to the criteria listed above. Any disagreement in quality assessment and data collection was discussed and solved by a third investigator acting as the referee.

Data were extracted from all the included studies by two independent reviewers. The name of the first author and the year of publication were used to identify the study. Details of the study samples (number in each group), interventions (use of erlotinib, as well as details of other treatments, including adjuvant chemotherapy) and outcomes [including OS, PFS, ORR and adverse events (AEs)] were extracted. The data were extracted directly from the text or calculated from available information. Whenever reports pertained to sets of patients that overlapped, only the report with the longest follow-up (having the largest number of events) was used in the final analysis.

A total of seven criteria were used to evaluate the quality of included studies: i) whether the allocation method was completely random, ii) whether there was proper concealment of allocation, iii) whether there was equality between the two groups at the baseline in terms of prognostic features, iv) whether the eligibility criteria were described, v) whether blinding of the outcome assessors was performed, vi) whether loss to follow-up in each treatment group was demonstrated and vii) whether intention-to-treat analysis was considered. A total of seven or six items were required for a study to be rated as high quality, five or four items for fair quality and three or fewer for low quality (22).

### Statistical analysis

Outcomes of the included studies were integrated using the Review Manager 5.12 software provided by the Cochrane Collaboration (dowloaded from http://www.cochrane.org). Dichotomous clinical outcomes were reported as risk ratio (RR) or odds ratio (OR), and time to event data as HR (23). The corresponding 95% confidence interval (CI) was calculated, P<0.05 was considered to indicate a statistically significant result.

The χ^2^ test was used to test the heterogeneities of treatment effects between studies, P<0.10 was considered to indicate a statistically significant result. Moreover, the *I^2^* method was used to estimate total variations across studies that were due to heterogeneity rather than chance in percentage (25% was considered to indicate low-level heterogeneity, 25–50% as moderate and >50% as high) (24).

The pooled statistics were first calculated with a fixed effects model. For dichotomous variables, the Cochran-Mantel-Haenszel test was used to analyze the significance, while for survival data, a pooled estimate of the HR was computed according to the inverse-variance method with P<0.05 being considered to indicate a statistically significant result. If statistical heterogeneity was identified, one of the following techniques was used to explain it: i) random effects model that provides a more conservative analysis, ii) subgroup analyses including patients stratified by EGFR immunohistochemistry status (positive, negative) and smoking history (current, former, ever, non-smokers) or iii) sensitivity analyses.

To assess the possibility of publication bias, we performed the funnel plot test described by Egger *et al* (25). When the pooled results were significant, the number of patients needed to treat (NNTs) were calculated by pooling absolute risk differences in studies included in our meta-analyses (26–28). For all the analyses, the forest plots were generated to exhibit results with point estimates and 95% CIs for each study and the overall size of the squares is proportional to effect size.

## Results

### Studies included

**Figure 1:**
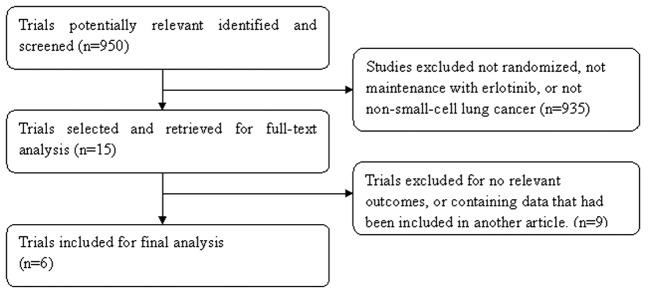
Flow diagram of study selection.

The study selection process was summarized, as recommended by the QUOROM statement (Fig. 1) (29). In total, 950 studies were identified and screened. Subsequently, selection was performed according to the inclusion/exclusion criteria described and 935 items were excluded at the primary selection step by browsing the retrieved titles and their abstracts. At the secondary selection step, nine studies were further excluded after reading the full text of 15 potentially eligible studies. Finally, six studies comprising 4,372 patients with histologically proven NSCLC met the inclusion criteria. Of these studies, four were published in full text (16,20,21,30), while the other two were reported in the annual meeting of the ASCO in the form of abstracts (31,32). There were certain differences in the experimental designs. Of the included studies, two administered erlotinib concurrently with chemotherapy followed by a maintenance phase (20,21), another study adopted a sequential regimen of erlotinib and chemotherapy (30), while the remaining three studies continued with erlotinib after chemotherapy (16,31,32). The main characteristics of these studies and the evaluations of the studies are presented in Tables I and II, respectively. A total of 4,327 patients were available for analysis and 2,191 patients were randomized to maintain with erlotinib (treatment group).

### Analysis of efficacy

**Figure 2:**
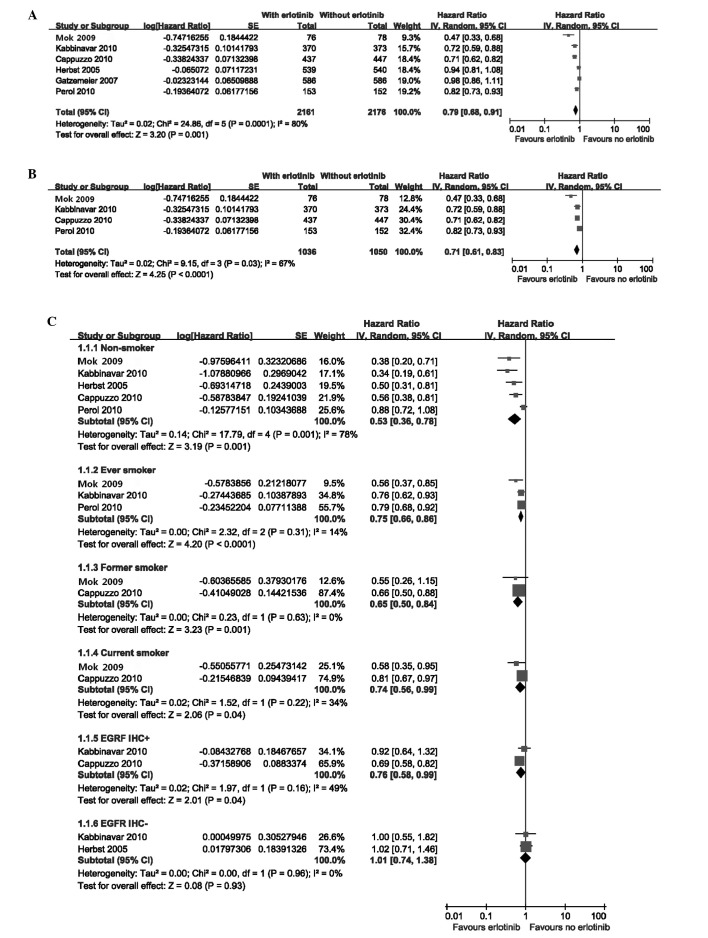
(A) Comparative effect of progression-free survival of maintenance with erlotinib vs. control. (B) Comparative effect of progression-free survival of maintenance with erlotinib vs. control after excluding the two studies using erlotinib concurrent with chemotherapy. (C) Subgroup analyses in progression-free survival of maintenance with erlotinib vs. control, stratified by EGFR status (positive, negative) and smoking history (current, former, ever, non-smokers). IHC+, immunohistochemistry-positive; IHC−, immunohistochemistry-negative.

The meta-analysis showed a longer PFS in patients who received erlotinib as maintenance therapy [random effects: HR= 0.79 (95% CI= 0.68–0.91); P= 0.001; NNT=5], showing a high heterogeneity level [χ^2^=24.86, df=5 (P=0.0001); *I^2^*=80%] (Fig. 2A). To further explore this heterogeneity, we excluded the two studies that used erlotinib concurrently with chemotherapy (20,21). The benefit to PFS was sustained [random effects: HR=0.71 (95% CI=0.61–0.83); P<0.001; NNT=3] with no significant change in the heterogeneity [χ^2^=9.15, df=3 (P=0.003); *I^2^*=67%] (Fig. 2B). Planned subgroup analyses also suggested that the PFS benefit with erlotinib was consistent across the majority of clinical subgroups with the exception of the EGFR immunohistochemistry-negative (EGFR IHC−) population. In addition, smokers did not obtain the greatest benefit from erlotinib [random effects: HR=0.53 (95% CI=0.36–0.78); P=0.001; NNT=2] (Fig. 2C).

**Figure 3:**
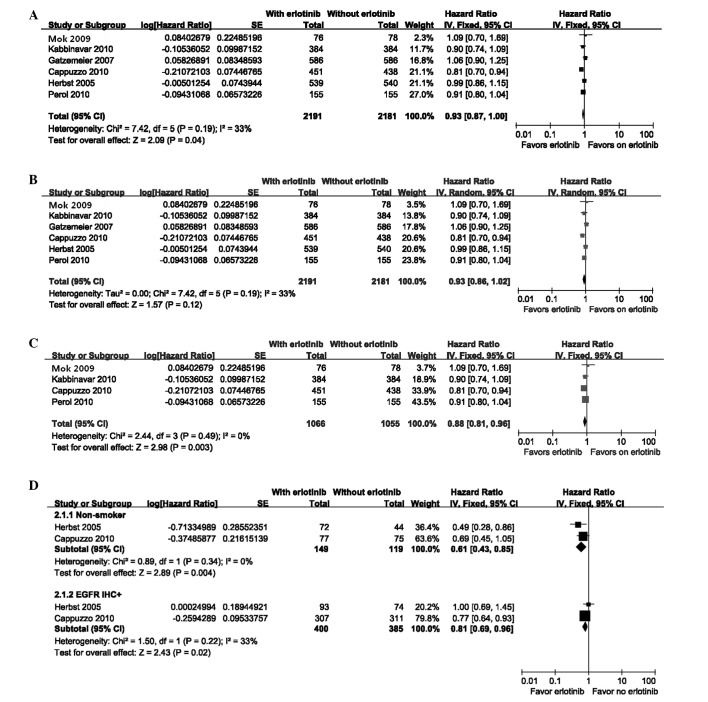
(A) Comparative effect of overall survival of maintenance with erlotinib vs. control using fixed effects model. (B) Comparative effect of overall survival of maintenance with erlotinib vs. control using random effects model. (C) Comparative effect of overall survival of maintenance with erlotinib vs. control after excluding the two studies using erlotinib concurrent with chemotherapy. (D) Subgroup analyses in overall survival of maintenance with erlotinib vs. control for non-smokers and the immunohistochemistry-positive (IHC+) patients.

The OS was slightly longer for patients who received erlotinib as maintenance therapy [fixed effect: HR= 0.93 (95% CI=0.87–1.00); P=0.04; NNT=15] with moderate heterogeneity [χ^2^=7.42, df=5 (P=0.19); *I^2^*=33%] (Fig. 3A). However, the random effects model indicated no significant difference [random effects: HR= 0.93 (95% CI= 0.86–1.02); P= 0.12] (Fig. 3B). When the two studies were excluded (20,21), due to concurrent erlotinib treatment and chemotherapy, the heterogeneity disappeared [χ^2^=2.44, df=3 (P=0.49); *I^2^*= 0%]. The treatment group obtained a greater benefit to OS compared with that in the control group [fixed effects: HR= 0.88 (95% CI= 0.81–0.96); P= 0.003; NNT=8] (Fig. 3C). Due to limited information, there were only two subgroups available for subset analyses for OS and the results are presented in Fig. 3D.

**Figure 4:**
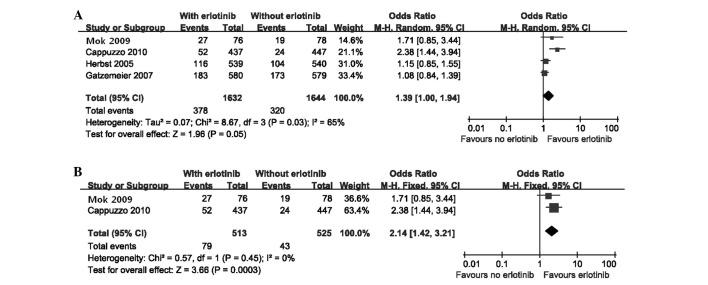
(A) Comparative effect of the objective response rates of maintenance with erlotinib vs. control. (B) Comparative effect of the objective response rates of maintenance with erlotinib vs. control after excluding the two studies using erlotinib concurrent with chemotherapy.

The meta-analysis of ORR is presented in Fig. 4A. The random effects model analysis indicated that there was no significant difference in ORR between the erlotinib and control groups [random effects: OR=1.39; (95% CI=1.00–1.94); P= 0.05]. Due to evident heterogeneity [χ^2^=8.67, df=3 (P=0.03); *I^2^*=65%], we also performed a sensitivity analysis by excluding the two studies (20,21). The results demonstrated a higher ORR in patients who received erlotinib only after chemotherapy [fixed effect: OR=2.14; (95% CI=1.42–3.21); P=0.0003], with no heterogeneity [χ^2^=0.57, df=1 (P=0.45); *I^2^*=0%] (Fig. 4B).

### Analysis of safety

**Figure 5:**
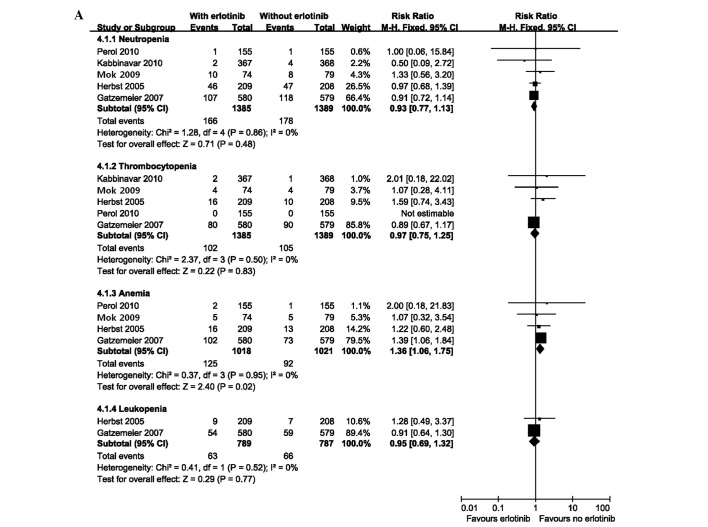

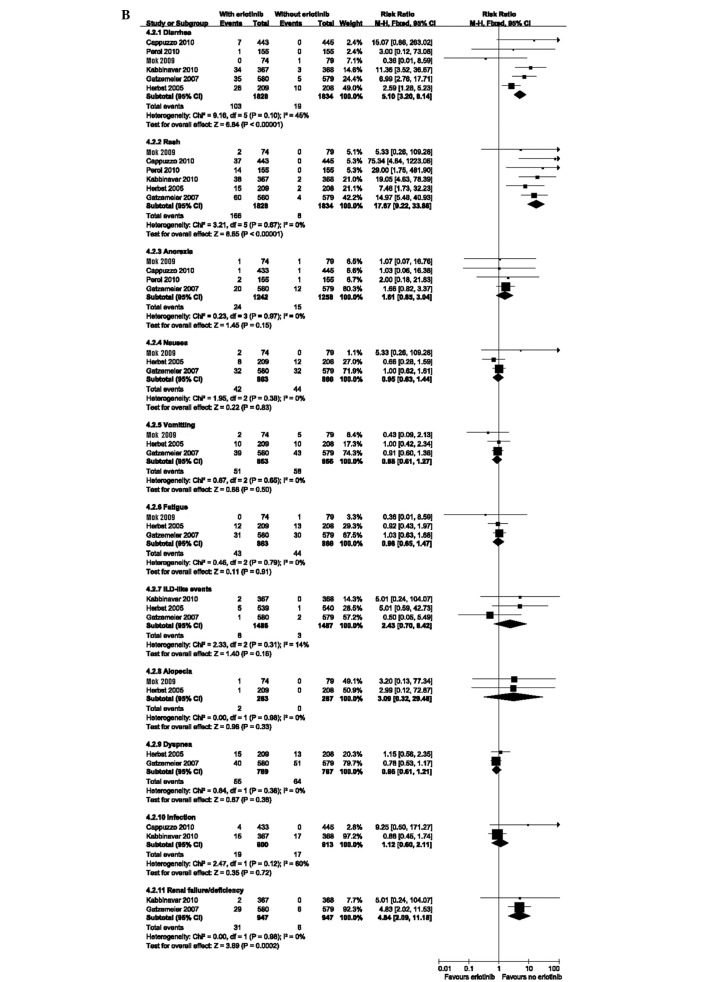

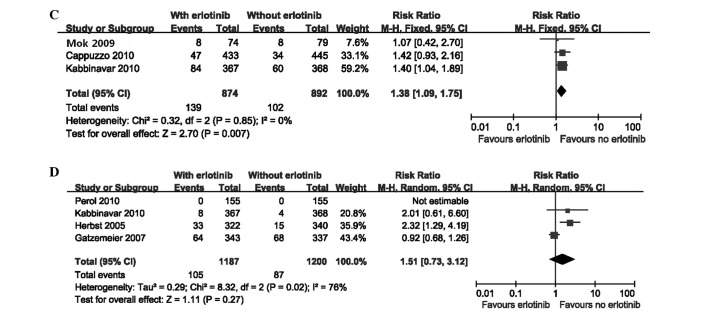
(A) Comparative effect of hematological toxicities of maintenance with erlotinib vs. control. (B) Comparative effect of non-hematological toxicities of maintenance with erlotinib vs. control. ILD, interstitial lung disease. (C) Comparative effect of serious adverse events of maintenance with erlotinib vs. control. (D) Comparative effect of treatment-related deaths of maintenance with erlotinib vs. control.

Pooled safety analyses of reported grade three or four AEs of interest, serious adverse events (SAEs) and treatment-related deaths were performed. The group receiving erlotinib had a higher incidence of anemia [fixed effect: RR=1.36; (95% CI=1.06–1.75); P=0.02]. No difference was observed in patients with other hematological toxicities including neutropenia, thrombocytopenia and leukopenia (Fig. 5A). With regard to the non-hematological toxicities, patients receiving erlotinib experienced a significantly higher incidence of diarrhea, skin toxicity and renal impairment with a pooled HR of 5.10 [fixed effect: (95% CI=3.20–8.14); P<0.00001], 17.67 [fixed effect: (95% CI=9.22–33.86); P<0.00001] and 4.84 [fixed effect: (95% CI=2.09–11.18); P=0.0002], respectively (Fig. 5B). Although the SAEs occurred more frequently in the experimental group [fixed effect: RR=1.38 (95% CI=1.09–1.75); P=0.007] (Fig. 5C), there was no significant difference in the incidence of treatment-related deaths [fixed effect: RR=1.51 (95% CI= 0.73–3.12); P= 0.27] (Fig. 5D). The funnel plot test did not reveal a significant publication bias (Egger test; P>0.05).

## Discussion

At present, whether maintenance therapy is necessary for advanced NSCLC remains controversial. It is also unclear which population of patients may gain the greatest benefit from this new approach. In the present study, we examined the efficacy and safety of maintenance with erlotinib for unresectable NSCLC. As shown by the meta-analysis, PFS was greatest with maintenance with erlotinib for patients with NSCLC. In the OS analysis, we observed a small increase in the maintenance group in a fixed effects model with a moderate-level heterogeneity. When a random effects model analysis was performed, this difference disappeared. There was no significant difference in ORR between the two groups in the original analysis. To explain the causes of heterogeneity among these studies, we conducted sensitivity and subgroup analyses. Preclinical evidence suggested that there may be a potential antagonism between the constituents of the combination therapy. EGFR TKIs induce G_1_ phase cell cycle arrest, which protects cells from the cytotoxic effects of cell-cycle phase-dependent chemotherapeutic agents, while alternative dosing schedules, including sequential or pulse dosing of erlotinib, proved to be more effective than concurrent administration (33,34). Based on these observations, the sensitivity analysis was performed by excluding the two studies that administered erlotinib concurrently with chemotherapy before the maintenance phase (20,21). The results showed the benefit to PFS was sustained, and maintenance erlotinib sequential with or just after first-line chemotherapy may improve OS and ORR. This fact indirectly proved that the concurrent administration of erlotinib and chemotherapy is not suitable for patients with NSCLC and may counteract the effect of maintenance therapy. By contrast, a sequential schedule has been successfully used to avoid the potential cell-cycle-based antagonism between EGFR TKIs and chemotherapy (30). However, the optimal sequential schedule of erlotinib with chemotherapy remains unclear.

It is worth noting that although erlotinib in combination with chemotherapy showed no benefit to survival compared with the placebo group for all the patients in the two excluded studies, the non-smoker subgroup experienced substantial prolongation in survival associated with erlotinib. There was no apparent pharmacokinetic interaction between erlotinib and cytotoxic drugs (20,21). The subset analyses in our study also demonstrated that maintenance with erlotinib prolonged PFS vs. control across the majority of clinical subgroups stratified by EGFR status and smoking history, with the exception of patients with EGFR IHC−. This result contradicted the opinion that an unidentified negative interaction exists between targeted agents and chemotherapy. In mice-bearing human NSCLC xenografts, a combination of erlotinib with cytotoxic drugs produced additive or synergistic antitumor activity (35). This contradiction suggests that the exact interaction between erlotinib and chemotherapy remains unclear, and more large-scale randomized clinical trials and preclinical studies are required to reveal their potential associations. Therefore, it appears that administration of erlotinib only after completion of first-line chemotherapy rather than a combination regimen should be the optimal maintenance setting.

The pooled results of side-effects were almost consistent with other studies. There was a small toxicity increase in patients with erlotinib. The most common side-effects were diarrhea and rash, which were usually controllable. Renal impairment was also higher in the treatment group due to insufficient hydration as indicated by the authors (20), however, this was not reported in previous erlotinib studies. Due to the similar incidence of treatment-related mortalities in the two groups, we supposed that maintenance erlotinib in unresectable NSCLC is tolerated.

Based on the pooled analysis of a large number of patients, we concluded that maintenance with erlotinib immediately after chemotherapy for unresectable NSCLC patients is associated with significant improvement in PFS, OS and ORR, as compared with placebo or observation only. However, this meta-analysis has its limitations. Due to limited data, we failed to perform pooled analyses of quality-of-life and cost-effectiveness, which are useful for doctors to determine whether the involved patients should receive maintenance therapy or a ‘treatment holiday’. Subsequent therapy may affect the OS of patients, but this issue was not analyzed in the present study. In addition, the number of included studies is small with little difference in design and one study did not achieve the mature OS data (32).

In an era of individualized treatment, continuing RTCs are required to identify patients who may derive greater benefit from erlotinib in this setting, and to compare the efficacy of erlotinib used as maintenance therapy with second-line treatment. Due to the fast development of molecular biology, genotyping is likely to replace traditional histopathological classification in the future and is likely to be more effective in treatment prediction and more prognostic of survival rates of patients with advanced cancer. Therefore, gene expression profiling on microarrays, including EGFR-activating mutations should be investigated. Thus, this meta-analysis should be updated in the future to clarify the effects of erlotinib in patients with advanced NSCLC. The incremental cost and toxicity associated with the adoption of this regimen should also be evaluated.

## Figures and Tables

**Table I:** Characteristics of included studies.

Study	Design	n	Patients	Intervention	Outcomes
Herbst *et al* (21)	Multi-center, randomized placebo-controlled phase III trial	1079	CT-naive advanced (stage IIIB or IV) NSCLC	GP concurrent with Erl or placebo and followed by Erl or placebo	OS, TTP, ORR, safety, duration of response
Gatzemeier *et al* (20)	Multi-center, randomized placebo-controlled, double-blind, phase III trial	1172	CT-naive unresectable or recurrent or advanced (stage III or IV) NSCLC	PC concurrent with Erl or placebo and followed by Erl or placebo	OS, TTP, ORR, QOL, safety, duration of response
Mok *et al* (30)	Multi-center, randomized placebo-controlled phase II trial	154	Previously untreated advanced (stage IIIB or IV) NSCLC	Sequential Erl or placebo and CT, followed by Erl or placebo	NPR, RR, OS, PFS, safety, duration of response
Cappuzzo *et al* (16)	Multi-center, randomized placebo-controlled phase III trial	889	Unresectable or advanced (stage IIIB or IV) NSCLC	Maintenance Erl vs. placebo after 4 cycles of standard platinum-doublet CT	PFS, OS, safety, QOL
Perol *et al* (32)	Randomized, three group phase III trial	310	Stage IIIB or IV NSCLC	Maintenance Erl vs. Gem vs. observation after 4 cycles	PFS, OS, safety symptom control of GP
Kabbinavar *et al* (31)	Randomized, double-blind, placebo-controlled, phase IIIb trial	768	Previously untreated recurrent or advanced (stage IIIB or IV) NSCLC	Maintenance Erl plus Bev vs. after 4 cycles of first-line CT combined Bev	PFS, OS, safety

NSCLC, non-small cell lung cancer; CT, chemotherapy; GP, gemcitabine + cisplatin; PC, paclitaxel + carboplatin; Erl, erlotinib; Bev, bevacizumab; Gem, gemcitabin; RR, response rate; OS, overall survival; PFS, progression-free survival; TTP, time to progression; NPR, non-progression rate; QOL, quality of life.

**Table II:** Quality of included studies.

Study	Truly random	Random allocation	Equivalence of baseline features	Eligibility criteria	Blinding assessment	Loss to follow-up	Intent to treat	Study quality
Herbst *et al* (21)	Yes	Yes	Yes	Yes	Yes	Unclear	Yes	High
Gatzemeier *et al* (20)	Yes	Yes	Yes	Yes	Yes	Yes	Unclear	High
Mok *et al* (30)	Yes	Yes	Yes	Yes	Unclear	Yes	Yes	High
Cappuzzo *et al* (16)	Yes	Yes	Yes	Yes	Yes	Unclear	Yes	High
Perol *et al* (32)	Yes	No	Yes	Yes	Yes	Unclear	Yes	Fair
Kabbinavar *et al* (31)	Yes	Yes	Yes	Yes	Unclear	Unclear	Yes	Fair
